# EHMTI-0279. Deep brain stimulation for refractory chronic cluster headache

**DOI:** 10.1186/1129-2377-15-S1-C44

**Published:** 2014-09-18

**Authors:** S Miller, H Akram, S Lagrata, M Hariz, M Matharu, L Zrinzo

**Affiliations:** 1 Headache, UCL Institute of Neurology, London, UK; 2 Functional Neurosurgery, UCL Institute of Neurology, London, UK

## Introduction

Chronic cluster headache (CCH) is an excruciating, unilateral headache with recurrent episodes of severe pain associated with ipsilateral autonomic features. 10-20% of patients are refractory to medical management. We present a prospective cohort of 19 patients with intractable CCH treated with posterior hypothalamic deep brain stimulation (DBS).

## Methods

Patients with refractory CCH referred to multidisciplinary headache clinic at our centre underwent DBS. Clinical data was collected pre and post-treatment. Headache load (HAL) (defined as [severity (on the visual analogue score)] x [duration] x [frequency] of headaches over a 2 week period) was calculated before and after treatment. A treatment response was identified as a 30% or more reduction in HAL.

## Results

19 patients (M=15) with a median age of 48 years (33-67 years) underwent surgery. Median follow up time was 12 months (9-48 months). 17 patients had at least one year follow up. Five patients failed to respond to treatment but nine showed a reduction in HAL of more than 80%. Within three months of surgery, the median change in HAL was 62% (0-100%) and at twelve months was 69% (0-100%). Significant differences exist between HAL at baseline and at three (p=0.001) and twelve months (p=0.06). There were no serious adverse events. One patient reported persistent diplopia, which was due to decompensation of a long-standing third nerve palsy.

## Conclusions

Posterior Hypothalamic DBS appears a safe and effective treatment for CCH and should be considered for suitable patients who fail conventional treatment.

No conflict of interest.

